# Slit lamp- and noncontact lens-assisted photography: a novel technique for color fundus photograph-like fundus imaging

**DOI:** 10.1007/s10792-014-9993-x

**Published:** 2014-09-07

**Authors:** Yoshikatsu Hosoda, Akihito Uji, Nagahisa Yoshimura

**Affiliations:** Department of Ophthalmology and Visual Sciences, Kyoto University Graduate School of Medicine, 54 Shogoin Kawahara-cho, Sakyo-Ku, Kyoto, 606-8507 Japan

## Manuscript

Digital fundus photography, commonly used in the diagnosis and monitoring of many ocular diseases, is an essential part of ophthalmology practice. However, fundus cameras are often costly and rely on separate computers for viewing and transmission of fundus photographs. In addition, adoption of electronic clinical record systems is expected to increase the demand for simple and cost-effective alternatives to the fundus camera that will allow the doctors to capture fundus images themselves during a medical examination. We describe a novel modified technique using slit-lamp biomicroscopy with a noncontact lens, which enables simple and convenient color fundus imaging.

**Fig. 1:**
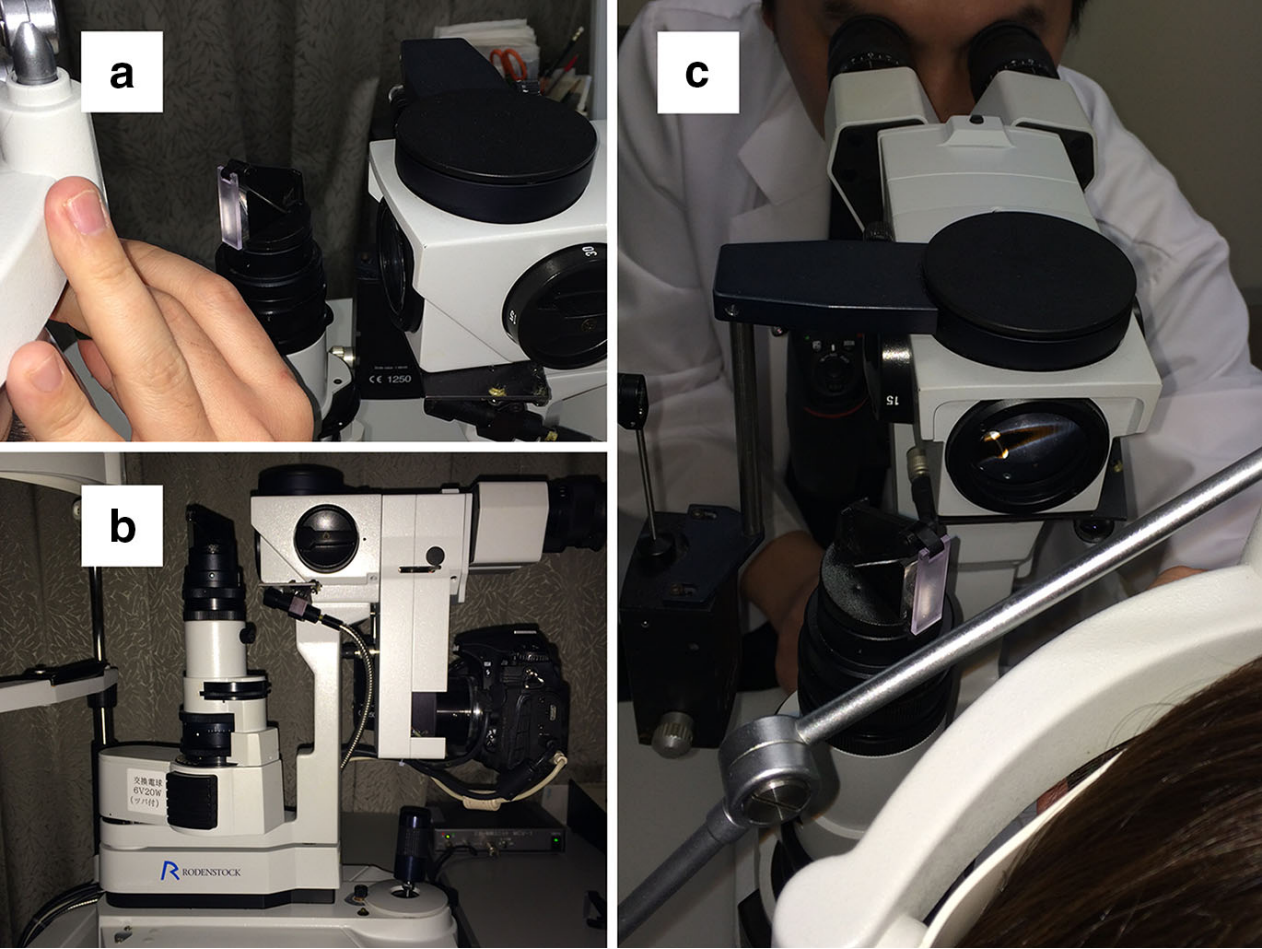
**a** Light diffuser placed on the slit lamp. **b** Digital single-lens reflex camera connected to the slit lamp. **c** Slit lamp with 90 D indirect lens used to capture a fundus image

We called our new technique slit lamp- and noncontact lens-assisted photography (SNAP). The technique is basically the same technique as conventional fundus imaging using slit-lamp biomicroscopy with a noncontact lens. However, SNAP images are obtained with a light diffuser on the slit lamp, and the slit lamp oriented at a relatively large angle of approximately 20° to 30° with respect to the optic axis (Fig. 1). SNAP images were captured with a digital single-lens reflex camera (Nikon D300 s, Nikon Imaging Japan Inc., Tokyo, Japan) connected to the slit lamp (RO 5000, Rodenstock, Munich, Germany) and a 90 D lens (Super Field NC lenses, Volk Optical Inc., Mentor, OH, USA).

**Fig. 2:**
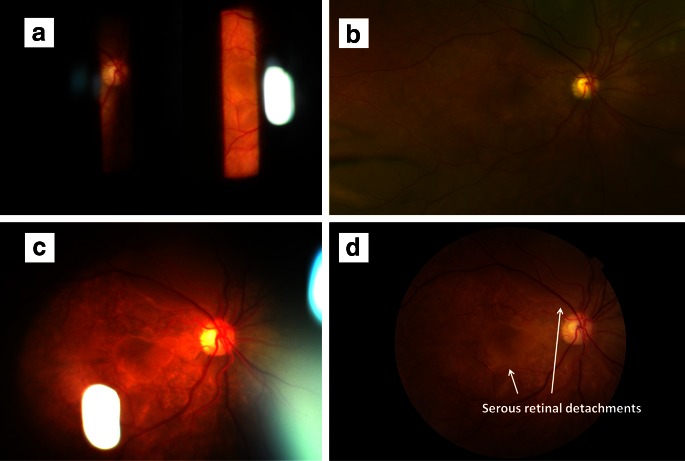

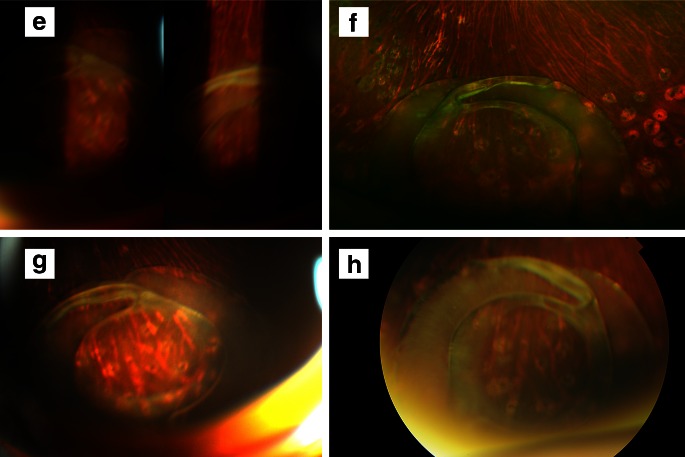
**a**, **b**, **c**, **d** Images from a patient with Vogt-Koyanagi-Harada disease. **e**, **f**, **g**, **h** Images from a patient with dropped intraocular lens on the retinal surface. **a**, **e** 90 D lens and slit lamp. **b**, **f** Optos 200Tx imaging system. **c**, **g** Our novel technique. **d**, **h** Conventional fundus camera

Color fundus photograph-like fundus images of the retina could be captured not only at the posterior pole but also at the peripheral part using the SNAP technique. The imaging fields of SNAP images were wider than those of basic slit-like fundus images and equivalent to those of color fundus photographs obtained with a conventional fundus camera (Fig. 2). Meanwhile, SNAP images displayed unnecessary reflection in the peripheral image.

## Comment

Use of a smartphone for fundus imaging [1–5] demonstrates the appeal of techniques for obtaining fundus images easily and quickly. SNAP, which requires only an ordinary camera, slit lamp, and noncontact lens, is a useful, easy, readily available, and cost-effective technique that enables frequent recording of fundus images in partly digitalized modern clinical practice. Possible future modalities to reduce these reflections include a lens with a coating that reduces reflections from its surface.
